# Domain Adaptation Using a Three-Way Decision Improves the Identification of Autism Patients from Multisite fMRI Data

**DOI:** 10.3390/brainsci11050603

**Published:** 2021-05-08

**Authors:** Chunlei Shi, Xianwei Xin, Jiacai Zhang

**Affiliations:** 1 School of Artificial Intelligence, Beijing Normal University, Beijing 100875, China; 201931210011@mail.bnu.edu.cn (C.S.); xinxianwei@mail.bnu.edu.cn (X.X.); 2 Engineering Research Center of Intelligent Technology and Educational Application, Ministry of Education, Beijing 100875, China

**Keywords:** autism spectrum disorder, machine learning, three-way decision, domain adaptation

## Abstract

Machine learning methods are widely used in autism spectrum disorder (ASD) diagnosis. Due to the lack of labelled ASD data, multisite data are often pooled together to expand the sample size. However, the heterogeneity that exists among different sites leads to the degeneration of machine learning models. Herein, the three-way decision theory was introduced into unsupervised domain adaptation in the first time, and applied to optimize the pseudolabel of the target domain/site from functional magnetic resonance imaging (fMRI) features related to ASD patients. The experimental results using multisite fMRI data show that our method not only narrows the gap of the sample distribution among domains but is also superior to the state-of-the-art domain adaptation methods in ASD recognition. Specifically, the ASD recognition accuracy of the proposed method is improved on all the six tasks, by 70.80%, 75.41%, 69.91%, 72.13%, 71.01% and 68.85%, respectively, compared with the existing methods.

## 1. Introduction

Autism spectrum disorder (ASD) is a common neurodevelopmental disease originating in infancy [1,2,3,4,5,6]. According to a recent study, one in 45 children in the world has autism, and the number of affected children has increased by 78% in the last decade [7]. Some symptoms of ASD even appear in young children by the age of two years [8]. Therefore, the early diagnosis of and intervention in ASD have received great attention in recent years [9,10]. Researchers have applied machine learning methods to identify biomarkers from resting-state functional magnetic resonance imaging (rs-fMRI) data to assist in diagnosing ASD [11,12,13].

Machine learning methods have demonstrated their effectiveness with the assumption that we have sufficient training data and test data drawn from the same distribution [14,15]. However, this assumption calling for enough examples is not always satisfied in practical applications and is not true in most cases, which will lead to the poor generalization ability of a model trained on one dataset when applied to another new dataset. First, clinical neural image datasets often face the problem of small dataset size due to their expensive acquisition and time-consuming labels. Therefore, multisite rs-fMRI data are often combined to expand the dataset in some research, such as ASD diagnosis, which leads to the second problem: samples from different scanners or acquisition protocols do not follow the same distribution in most cases [16,17].

The fMRI samples from different sites have also been named domains in the machine learning research community. In addition to the distribution difference of the training set (source domain) and the test set (target domain), the scarcity of labelled samples is another challenge to ASD recognition. Previous studies have investigated domain adaptation approaches to overcome site-to-site transfer [18]. Many studies have successfully applied domain adaptation to object recognition [19], activity recognition [20], speech recognition [21], text classification [22] and autism recognition [7]. The main goal of domain adaptation is to reduce the difference in the data distribution between the source domain and the target domain and then train a robust classifier for the target domain by reusing the labelled data in the source domain.

At present, the research on domain adaptation mainly focuses on three methods, namely, instance adaptation, feature adaptation and classifier adaptation. Specifically, the instance-based domain adaptation method reuses samples from the source domain according to a certain weighting rule. Instance adaptation has achieved good results by eliminating cross domain differences [23]. However, this method needs to satisfy two strict assumptions: (1) the source domain and the target domain follow the same conditional distribution, and (2) some data in the source domain can be reused in the target domain by reweighting. The classifier-based domain adaptation method transfers knowledge from the source domain to the target domain by sharing parameters between the source domain and target domain [24,25]. Classifier transfer has performed well with labelled samples. However, regarding ASD diagnosis from fMRI, the data distributions of different sites are different, and reliable labelled data are difficult to obtain.

Therefore, the application of domain adaptations based on instances or classifiers is relatively difficult. However, the feature-based domain adaptation method can learn the subspace geometrical structure [26,27,28,29] or distribution alignment [30,31,32]. This method gears the marginal or conditional distributions of different domains in a principled dimensionality reduction procedure. Our work employed the feature-based domain adaptation method to eliminate the divergence of the data distribution.

Currently, most feature-based domain adaptation research is devoted to the adaptation of the marginal distribution, conditional distribution, or both. For example, Long et al. [26] found that the marginal distribution and conditional distribution between domains are different in the real world, and better performance can be achieved if the two distributions are adapted simultaneously. Subsequently, some studies based on joint distribution adaptation have been proposed successively [33,34,35], and these works have greatly contributed to the development of domain adaptation.

It is worth noting that in order to obtain the pseudolabels of the target domain data, the traditional methods usually directly apply the classifier trained in the source domain to the prediction of the target domain data. However, these pseudolabels might lead to some error due to the possible domain mismatch. Here, we proposed a robust method using a three-way decision model derived from triangular fuzzy similarity. The proposed model roughly classified the samples in the target domain into three domains, i.e., the positive region, the negative region and the boundary region. Then, the label propagation algorithm was used to optimize the label and make secondary decisions on the boundary region samples. The experiments demonstrate that our method can effectively improve the classification performance for automated ASD diagnosis.

The contributions of this paper is as follows:

1. A three-way decision model based on triangular fuzzy similarity is proposed to reduce the cost loss of target domain data prediction. To the best of authors’ knowledge, it is the first time to combine the three-way decision model and the distribution adaptation method to reduce the distribution differences between domains. The proposed method extends the application of machine learning in the field of decision making.
2. Our method utilizes the label information from the source domain and the structural information from the target domain at the same time, which not only reduces the distribution differences between domains but also further improves the recognition ability of the target domain data.
3. Comprehensive experiments on the Autism Brain Imaging Data Exchange (ABIDE) dataset prove that our method is better than several state-of-the-art methods.

The remainder of this paper is organized as follows. Section 2 reviews the related work concisely. In Section 3, we elucidate the foundation of the proposed method. Our proposed method is illustrated in detail in Section 4. Then, the results and discussion are presented in Section 5 and Section 6, respectively. Finally, the paper is concluded in Section 7.

## 2. Related Work

It has been a lasting challenge to build maps between different domains in the field of machine learning. Domain adaptation has become a hot research topic in disease diagnosis with machine learning. In this paper, we proposed transfer learning based on distribution adaptation and three-way decisions. To elaborate the proposed method, we will introduce the related work from the following three aspects in this section.

### 2.1. Distribution Adaptation

Distribution adaptation is one of the most commonly used methods in domain adaptation. It seeks a space translation and eliminated data distribution differences between source and target domains by explicitly minimizing the predefined distance in this feature space. According to the nature of the data distribution, distribution adaptation can be divided into three categories: marginal distribution adaptation, conditional distribution adaptation and joint distribution adaptation.

Pan et al. [36] first proposed a transfer component analysis (TCA) method based on marginal distribution adaptation, which used the maximum mean discrepancy (MMD) to measure the distance between domains and achieve feature dimensionality reduction. The method assumes that there is a mapping so that the marginal distribution of the mapped source domain and target domain is similar in the new space. The disadvantage of TCA is that the algorithm only focuses on reducing the cross-domain marginal distribution difference without considering reducing the conditional distribution difference. Long et al. [37] proposed a transfer joint matching (TJM) method, which mainly combines source domain sample selection and distribution adaptation to further eliminate cross domain distribution differences.

Recently, in the work based on conditional distribution adaptation, Wang et al. [38] proposed a stratified transfer learning method (STL). Its main idea is to reduce the spatial dimension in the reproducing kernel Hilbert space (RKHS) by using the intraclass similarity so as to eliminate the distribution differences. However, in the real world, differences may exist in both marginal distributions and different conditional distributions. Adjusting only one of the distributions is insufficient to bridge domain differences. In order to solve this problem, Long et al. [26] proposed the joint distribution adaptation (JDA) method. The goal of JDA is to jointly adjust the marginal distribution and the conditional distribution using a principled dimensionality reduction process, and the representation in this common feature space reduced the domain differences significantly. Other work extended JDA by adding structural consistency [29], domain invariant clustering [30] and label propagation [31].

To provide supervised information for the target domain, JDA methods applied source domain classifiers in the target domain and took the classifier outputs as the pseudolabels of the target domain data. However, due to the different data distributions of domains, the direct use of these inaccurate pseudolabels will result in the degradation of the final model’s performance.

Considering the domain gap both in labels and samples, three-way decisions provided a novel method to transmit the label information between domains and reuse the intrinsic structural information of the target domain data to further improve the performance of the model in the domain adaptation process.

### 2.2. Three-Way Decisions

As an effective extension of traditional rough sets, three-way decision [39] (3WD) theory has been widely applied to address uncertain, inaccurate and fuzzy problems, such as medical diagnosis [40], image processing [41], emotion analysis [42], etc. In simple terms, 3WD divides the universe of discourse into three disjoint parts, i.e., the positive region (Pos), the negative region (Neg), and the boundary region (Bnd), through a pair of upper and lower, approximately. Acceptance and rejection decisions were made for the objects in Pos and Neg, respectively. Specifically, the objects in Bnd adopt the delay decision.

Strictly speaking, the current 3WD research can be divided according to whether it is based on decision-theoretic rough sets (DTRSs) [43]. For example, Zhang et al. [44] proposed a 3WD model for interval-valued DTRSs and gave a new decision cost function. Liu et al. [45] introduced intuitionistic fuzzy language DTRSs and 3WD models to obtain fuzzy information in uncertain languages. Agbodah [46] focused on the study of the DTRS loss function aggregation method in group decision making and utilized it to construct a 3WD model.

In addition, scholars have also conducted in-depth explorations on 3WD outside the DTRS framework. For example, Liang et al. [47] integrated the risk preference of decision makers into the decision-making process and proposed a 3WD model based on the TODIM (an acronym in Portuguese for interactive multicriteria decision making) method. Qian et al. [48] investigated three-way formal concept lattices of objects (properties) based on 3WD. Yang et al. [49] presented a 3WD model oriented to multigranularity space to adapt 3WD to intuitionistic fuzzy decisions.

From a broad perspective, 3WD can be classified as static or dynamic. Static 3WD includes related research based on the DTRS framework and fusion of other theories. Dynamic 3WD mainly addresses the problem of constantly changing data in time series and space, and its typical representative is the sequential 3WD model [50]. For example, Yang et al. [51] proposed a three-way calculation method for dynamic mixed data based on time and space. Zhang et al. [52] systematically investigated a new sequential 3WD model to balance autoencoder classification and reduce its misclassification cost. Liu et al. [53] combined 3WD and granular computing to construct a dynamic three-way recommendation model to reduce decision-making costs.

3WD theory has been widely used in many areas, such as emerging three-way formal concept analysis [54], three-way conflict analysis [55], three-way granular computing [56], three-way classification [57], three-way recommendation [58], and three-way clustering [59]. This paper will combine the idea of 3WD to improve the performance of heterogeneous ASD data diagnosis by reducing the difference in the data distributions between the source domain and target domain.

### 2.3. Application of Machine Learning in Identification of ASD Patients

In recent years, magnetic resonance imaging (MRI) has been widely used in clinical practice [60,61]. The commonly used MRI can be divided into structural MRI (sMRI) and functional MRI (fMRI). As fMRI can measure the hemodynamic changes caused by the activity of brain neurons, it has been widely used in the research of brain dysfunction diseases. For example, Li et al. [62] proposed a 4D deep learning model for ASD recognition that can utilize both temporal and spatial information of fMRI data. In the work of Riaz et al. [63], they proposed an end-to-end deep learning method called DeepfMRI for accurately identifying patients with Attention Deficit Hyperactivity Disorder (ADHD) and achieved an accuracy rate of 73.1% on open datasets. To study the relationship between mild cognitive impairment (MCI) and Small Vessel Disease (SVD), Diciotti et al. [64] applied the Stroop test to the rs-fMRI data of 67 MCI subjects and found that regional homogeneity of rs-fMRI is significantly correlated with measurements of the cognitive deficits.

As a neurodevelopmental disorder, early diagnosis of ASD is very important to improve the quality of life of patients. In recent years, researchers have attempted to extract biomarkers representing ASD from fMRI data using machine learning methods, so as to provide an auxiliary diagnosis for clinicians. For example, Lu et al. [65] proposed a multi-kernel-based subspace clustering algorithm for identifying ASD patients, which still has a good clustering effect on high-dimensional network datasets. Leming et al. [66] trained a convolutional neural network and applied it to ASD recognition, and their experiments showed that deep learning models that distinguish ASD from NC controls focus broadly on temporal and cerebellar connections. However, the problem of small size fMRI data prevented the generalization of the above research works [67].

To solve this problem, the Autism Brain Imaging Data Exchange, an international collaborative project, has collected data from over 1000 subjects and made the whole database publicly available. Based on the ABIDE database, many advanced machine learning models have been proposed for the identification of ASD patients. For example, Eslami et al. [68] used autoencoder and single-layer perceptron to diagnose ASD and proposed a deep learning framework called ASD-DiagNet, which achieved classification accuracy of 70.3%. Bi et al. [69] used randomized support vector machine (SVM) clusters to distinguish ASD patients from normal controls and identified a number of abnormal brain regions that contribute to ASD. Mladen et al. [70] selected 368 ASD patients and 449 normal controls from ABIDE database, and then used the Fisher score as the feature selection method to quantitatively analyze 817 subjects and obtained classification accuracy of 85.06%.

## 3. Preliminaries

We start with the definition of the problem and the terms and introduce the notation we will use below. The source domain data denoted as $X_{s}\in {\mathbb{R}}^{d\times n_{s}}$ are drawn from distribution $P_{s}(X_{s})$, and the target domain data denoted as $X_{t}\in {\mathbb{R}}^{d\times n_{t}}$ are drawn from distribution $P_{t}(X_{t})$, where d is the dimension of the data instance and $n_{s}$ and $n_{t}$ are the number of samples in the source and target domains, respectively.

Assume a labelled source domain $D_{s}={\left\{(x_{i},y_{i})\right\}}_{{}_{i=1}}^{{}^{n_{s}}},\mathrm{where}\;x_{i}\in {\mathbb{R}}^{d\times n_{s}}$, and an unlabeled target domain $D_{t}={\left\{(x_{j})\right\}}_{{}_{j=1}}^{{}^{n_{t}}}\;\mathrm{and}\;x_{j}\in {\mathbb{R}}^{d\times n_{t}}$. We assume that their feature space and label space are the same, i.e., $X_{s}=X_{t}\;\mathrm{and}\;Y_{s}=Y_{t}$, but their marginal distribution and conditional distribution are different, i.e., $P_{s}(X_{s})\neq P_{t}(X_{t})\;\mathrm{and}\;P_{s}(Y_{s}|X_{s})\neq P_{t}(Y_{t}|X_{t})$.

Domain adaptation methods often seek to reduce the distribution differences across domains by explicitly adapting both the marginal and conditional distributions between domains. To be specific, domain adaptation seeks to minimize the distance (Equation (1)):

(1) $$D\left(D_{s},D_{t}\right)\approx D\left(P_{s}(X_{s}),P_{t}(X_{t})\right)+D\left(P_{s}(Y_{s}|X_{s}),P_{t}(Y_{t}|X_{t})\right)$$

where $D\left(P_{s}(X_{s}),P_{t}(X_{t})\right)$ and $D\left(P_{s}(Y_{s}|X_{s}),P_{t}(Y_{t}|X_{t})\right)$ are the marginal distribution distance and conditional distribution distance between domains, respectively.

There are many metrics that can be used to estimate the distance between distributions, such as the Kullback–Leibler (KL) divergence. However, most of these distance metrics are based on parameters, and it is difficult to calculate the distance. Therefore, Borgwardt et al. [71] proposed a nonparametric distance metric MMD using a kernel learning method to measure the distance between two distributions in RKHS. The definition of the MMD is as follows:

**Definition** **1.** *Given two random variables* $X_{s}$*and*$X_{t}$*, their MMD squared distance is calculated as follows (Equation (2)):*

(2) $$Dist\left(X_{s},X_{t}\right)=||\frac{1}{n_{s}}\sum_{i=1}^{n_{s}}\emptyset \left(x_{i}\right)-\frac{1}{n_{t}}\sum_{j=1}^{n_{t}}\emptyset \left(x_{j}\right)|{|}^{2}{}_{ℋ}$$

*where*ℋ*is a universal RKHS[72], and*∅:X→ℋ.

Next, we introduce the concepts of triangular fuzzy numbers and three-way decisions.

**Definition** **2.** *[73]. Let*$\tilde{t}=[t^{L},t^{M},t^{T}]$*be a triangular fuzzy number, where*$t^{L}$*and*$t^{T}$*denote the upper bound and lower bound of*$\tilde{t}$*, respectively, and*$t^{M}$*is the median of*$\tilde{t}$*. If*$0<t^{L}\leq t^{M}\leq t^{T}$*is satisfied, then*$\tilde{t}$* is called a normal triangular fuzzy number. For any two triangular fuzzy numbers*$\tilde{t}=[t^{L},t^{M},t^{T}]$*and*$\tilde{k}=[k^{L},k^{M},k^{T}]$, the distance between them is as follows (Equation (3)):

(3) $$d(\tilde{t},\tilde{k})=\sqrt{\frac{{\left(t^{L}-k^{L}\right)}^{2}+{\left(t^{M}-k^{M}\right)}^{2}+{\left(t^{T}-k^{T}\right)}^{2}}{3}}$$

In addition, the basic operations between $\tilde{t}=[t^{L},t^{M},t^{T}]$ and $\tilde{k}=[k^{L},k^{M},k^{T}]$ are as follows (Equation (4)):

(4) $$\begin{array}{l} \tilde{t}+\tilde{k}=[t^{L},t^{M},t^{T}]+[k^{L},k^{M},k^{T}]=[t^{L}+k^{L},t^{M}+k^{M},t^{T}+k^{T}]\\ \tilde{t}-\tilde{k}=[t^{L},t^{M},t^{T}]-[k^{L},k^{M},k^{T}]=[t^{L}-k^{L},t^{M}-k^{M},t^{T}-k^{T}]\\ \tilde{t}\times \tilde{k}=[t^{L},t^{M},t^{T}]\times [k^{L},k^{M},k^{T}]=[t^{L}\times k^{L},t^{M}\times k^{M},t^{T}\times k^{T}] \end{array}$$

**Definition** **3.** *[74]. Let U be the universe of discourse,*∀X∈U*. If threshold*0≤β<α≤1 exists, then its positive region, negative region and boundary region are defined with threshold (α,β) (Equation (5)):

(5) $$\begin{array}{l} Pos_{\left(\alpha ,\beta \right)}(X)=\{x\in U|\mathrm{Pr}(X|[x])\geq \alpha \}\\ Bnd_{{}_{\left(\alpha ,\beta \right)}}(X)=\{x\in U|\beta <\mathrm{Pr}(X|[x])<\alpha \}\\ Neg_{{}_{\left(\alpha ,\beta \right)}}(X)=\{x\in U|\mathrm{Pr}(X|[x])\leq \beta \} \end{array}$$

*where* [x] *is the equivalence class containing* x*, and* Pr(X|[x]) is the conditional probability.

## 4. Methods

### 4.1. Joint Distribution Adaptation

Domain adaptation seeks an invariant feature expression for the source domain and the target domain in a low-dimensional (K < d) space. Let $W\in {\mathbb{R}}^{d\times k}$ be the linear transformation matrix and $Z_{s}=W^{T}X_{s}$ and $Z_{t}=W^{T}X_{t}$ be the projected variables from the source and target data, respectively. We use the nonparametric metric MMD, which computes the distance between the sample means of the source and target data in the k-dimensional embeddings, to estimate the difference between distributions. Specifically, according to Equation (2), $D\left(P_{s}(X_{s}),P_{t}(X_{t})\right)$ can be expressed as (Equation (6)):

(6) $$D\left(P_{s}(X_{s}),P_{t}(X_{t})\right)=||\frac{1}{n_{s}}\sum_{i=1}^{n_{s}}W^{T}x_{i}-\frac{1}{n_{t}}\sum_{j=1}^{n_{t}}W^{T}x_{j}|{|}^{2}$$

By further using the matrix transformation rule and regularization and then minimizing the marginal distribution distance, Equation (6) can be formalized as follows (Equation (7)):

(7) $$D\left(P_{s}(X_{s}),P_{t}(X_{t})\right)=tr\left(A^{T}XM_{0}X^{T}A\right)$$

where X represents the input matrix containing $X_{s}$ and $X_{t}$. In addition, following [26], $M_{0}$ is the MMD matrix and can be constructed as follows (Equation (8)):

(8) $${\left(M_{0}\right)}_{ij}=\left\{ \begin{array}{cc} \frac{1}{n_{s}{}^{2}}, & x_{i},x_{j}\in D_{s}\\ \frac{1}{n_{t}{}^{2}}, & x_{i},x_{j}\in D_{t}\\ -\frac{1}{n_{s}n_{t}}, & otherwise \end{array}\right.$$

However, the label information of the domain data is not considered, which will lead to the lack of sufficient discriminability of the adapted features; therefore, so it is insufficient to adapt to the marginal distribution only. To solve this problem, we will next adjust the conditional distribution between domains.

Since no label information is available in the target domain, we cannot directly estimate the conditional distribution $P_{t}(Y_{t}|X_{t})$ of the target domain. Here, based on the concept of sufficient statistics, we can replace $P_{t}(Y_{t}|X_{t})$ and $P_{s}(Y_{s}|X_{s})$ with class conditional distributions $P_{t}(X_{t}|Y_{t})$ and $P_{s}(X_{s}|Y_{s})$, respectively. However, obtaining target domain label information through source domain data while reducing the distribution difference between domains is a challenging problem in unsupervised domain adaptation. In Section 4.2, we introduce how to obtain the label information of the target domain data so as to obtain the above class conditional distribution. Thus far, we can match the class condition distribution of the two domains. Similar to the calculation of the marginal distribution, we use the modified MMD formula to estimate the conditional distribution $D\left(P_{s}(Y_{s}|X_{s}),P_{t}(Y_{t}|X_{t})\right)$ between domains. $D\left(P_{s}(Y_{s}|X_{s}),P_{t}(Y_{t}|X_{t})\right)$ can be represented as (Equation (9)):

(9) $$D\left(P_{s}(Y_{s}|X_{s}),P_{t}(Y_{t}|X_{t})\right)=\sum_{c=1}^{C}||\frac{1}{n_{s}^{c}}\sum_{x_{i}\in D_{s}^{\left(c\right)}}^{}W^{T}x_{i}-\frac{1}{n_{t}^{c}}\sum_{x_{j}\in D_{t}^{\left(c\right)}}^{}W^{T}x_{j}|{|}^{2}$$

where c∈{1,2,3,⋅⋅⋅,C} is the class label, and $D_{s}^{\left(c\right)}$ and $D_{t}^{\left(c\right)}$ are samples belonging to class c in the source domain and target domain, respectively. $n_{s}^{c}$ and $n_{t}^{c}$ are the number of samples belonging to class c in the source domain and target domain, respectively.

Similar to the marginal distribution, we formalize Equation (9) as Equation (10) by using matrix transformation rules and regularization:

(10) $$D\left(P_{s}(Y_{s}|X_{s}),P_{t}(Y_{t}|X_{t})\right)=tr\left(W^{T}XM_{c}X^{T}W\right)$$

where the MMD matrices $M_{c}$ containing class labels are constructed as follows (Equation (11)):

(11) $${\left(M_{c}\right)}_{ij}=\left\{ \begin{array}{cc} \frac{1}{{\left(n_{s}^{c}\right)}^{2}}, & x_{i},x_{j}\in D_{s}^{\left(c\right)}\\ \frac{1}{{\left(n_{t}^{c}\right)}^{2}}, & x_{i},x_{j}\in D_{t}^{\left(c\right)}\\ -\frac{1}{n_{s}^{c}n_{t}^{c}}, & \left\{ \begin{array}{l} x_{i}\in D_{s}^{\left(c\right)},x_{j}\in D_{t}^{\left(c\right)}\\ x_{i}\in D_{t}^{\left(c\right)},x_{j}\in D_{s}^{\left(c\right)} \end{array}\right.\\ 0, & otherwise \end{array}\right.$$

In order to reduce both the marginal distribution and conditional distribution between domains, we incorporate Equations (7) and (10) into one object Function (Equation (12)):

(12) $$\begin{array}{l} \min\;\sum_{c=0}^{C}tr\left(W^{T}XM_{c}X^{T}W\right)+\lambda ||W|{|}^{2}{}_{F}\\ s.t.\;W^{T}XHX^{T}W=I \end{array}$$

where the first term considers both the adaptive marginal distribution and conditional distribution, and the second term is the regularization term. $||\cdot |{|}^{2}{}_{F}$ is the Frobenius norm, and λ is the regularization parameter. As noted in [29], adding the constraint in Function (12) would preserve the inner properties of the original data, which implies and introduces an additional data discrimination ability into the learned model. In addition, in function (12), X represents the input matrix containing $X_{s}$ and $X_{t}$; $I\in {\mathbb{R}}^{\left(n_{s}+n_{t})\times (n_{s}+n_{t}\right)}$ denotes the identity matrix; and $H=I-\frac{1}{n_{s}+n_{t}}1$ is the centering matrix, where 1 is the $\left(n_{s}+n_{t}\right)\times \left(n_{s}+n_{t}\right)$ matrix of ones.

To obtain the transformation matrix W, we obtain the Lagrange solution to function (12), which is rewritten as (Equation (13)):

(13) $$L=\sum_{c=0}^{C}tr\left(W^{T}XM_{c}X^{T}W\right)+\lambda ||W|{|}^{2}{}_{F}+tr\left(\left(I-W^{T}XHX^{T}W\right)\Phi \right)$$

where $\Phi =\left(\emptyset_{1},\emptyset_{2}\ldots \cdots ,\emptyset_{d}\right)$ is the Lagrange multiplier. Setting $\frac{\partial L}{\partial W}=0$, the original optimization problem is transformed into the following eigen-decomposition problem (Equation (14)):

(14) $$\left(\sum_{c=0}^{C}XM_{c}X^{T}+\lambda I\right)W=XHX^{T}W\Phi$$

The transformation matrix W is the solution to Equation (14) and thus builds the bridge between the source and target domains in the new expression $Z=\left(Z_{s},Z_{t}\right)$.

### 4.2. Three-Way Decision Model Based on Triangular Fuzzy Similarity

In practice, the conditional distribution cannot be obtained directly because there is no label information in the target domain. In order to solve this problem, we first give the concept of the degree of information difference and apply it to the construction of triangular fuzzy numbers and the calculation of the corresponding triangular fuzzy similarity. Then, according to the degree of association of the triangular fuzzy similarity between objects in the target domain, the target domain is divided into positive regions, negative regions and boundary regions with structural information.

For the convenience of the description, suppose that both the domain of discourse *U* and attribute set *A* are nonempty finite sets and that $x_{i}$ is an object in *U*, $a_{j}$ is an attribute in *A*, where i=1,2,⋯,n and j=1,2,⋯,m.

#### 4.2.1. Information Difference Degree and Triangular Fuzzy Similarity

**Definition** **4.** *Let*$U=\{x_{1},x_{2},\cdots ,x_{n}\}$*be the domain of discourse,*$A=\{a_{1},a_{2},\cdots ,a_{m}\}$*be the set of attributes, and the value of object*$x_{i}$*under attribute*$a_{j}$*be*$x_{ij}$*. When*$\forall a_{j},a_{k}\in A$*, the degree of information difference of object*$x_{i}$ is as follows (Equation (15)):

(15) $$ID_{i}(a_{j},a_{k})=\frac{1}{\exp(-\log_{2}{}^{\frac{\left|x_{ij}-x_{ik}\right|}{x_{ij}+x_{ik}}})}$$

**Remark** **1.**

*(1)*  *The greater the value of* $ID_{i}(a_{j},a_{k})$*is, the greater the degree of information difference of object*$x_{i}$*under*$a_{j}$*and*$a_{k}$*. When object*$x_{i}$*has the same description*$x_{ij}=x_{ik}=0$*for*$a_{j}$*and*$a_{k}$*, the real part of the log function will have a denominator of 0, i.e.,*$x_{ij}+x_{ik}=0$*. In this case, since*$|x_{ij}-x_{ik}|=0$*, we can obtain that the final degree of information deviation*$ID_{i}(a_{j},a_{k})$*is independent of the value of*$x_{ij}+x_{ik}$*. For the reasonableness of the calculation, let*$\frac{\left|x_{ij}-x_{ik}\right|}{x_{ij}+x_{ik}}=0$.
*(2)*  *For the convenience of the representation, we obtain the information difference matrix of object* $x_{i}$, which can be expressed as follows (Equation (16)):
  (16)                 a1                 a2           ⋯      am−1          amIDi=a1a2⋮am−1am[IDi11 IDi21 ⋯ IDi(m−1)1 IDim1IDi12 IDi22 ⋯ IDi(m−1)2 IDim2⋮ ⋮ ⋱ ⋮ ⋮IDi1(m−1) IDi2(m−1)⋯ IDi(m−1)(m−1) IDim(m−1)IDi1m IDi2m ⋯ IDi(m−1)m IDimm]
  *where*$ID_{i}^{jk}=ID_{i}(a_{j},a_{k})$*represents the degree of information difference of object*$x_{i}$*under attributes*$a_{j}$*and*$a_{k}$.

**Theorem** **1.** *According to definition 4, we have the following conclusions:*

*(1)*  *Boundedness:*$0\leq ID_{i}(a_{j},a_{k})\leq 1$.
*(2)*  *Monotonicity: The degree of information difference of*$x_{i}$*about*$a_{j}$*and*$a_{k}$ increases monotonously as the difference increases.
*(3)*  *Symmetry:*$ID_{i}(a_{j},a_{k})=ID_{i}(a_{k},a_{j})$.

**Proof.**  Properties (2) and (3) are easily proven by Definition 4.

(1) According to Definition 4, $\forall a_{j},a_{k}\in A$ and $x_{i}\in U$. When the description of $x_{i}$ under $a_{j}$ and $a_{k}$ appears in two extreme cases, namely, $x_{ij}=0\;\mathrm{and}\;x_{ik}=1$ or $x_{ij}=1\;\mathrm{and}\;x_{ik}=0$, we can obtain $|x_{ij}-x_{ik}|=1$, and the information difference reaches the maximum at this time, $ID_{i}(a_{j},a_{k})=1$. □

**Definition** **5.** *Let U be the domain of discourse, and the triangular fuzzy number of* $x_{i}$*under attribute set A is*${\tilde{x}}_{i}=[x_{i}{}^{L},x_{i}{}^{M},x_{i}{}^{T}]$*, where*$x_{i}{}^{L}=\min\{\sum ID_{i}^{jk}\}$*,*$x_{i}{}^{T}=\max\{\sum ID_{i}^{jk}\}$*,*$x_{i}{}^{M}=\max\{\sum \left|ID_{i}^{jk}\right|\}$*and*$\left|ID_{i}^{jk}\right|$*denotes the number of information difference values*$ID_{i}^{jk}$*. Then, the degree of triangular fuzzy similarity between*${\tilde{x}}_{i}$*and*${\tilde{x}}_{k}$ is as follows (Equation (17)):

(17) $${\tilde{S}}_{TF}({\tilde{x}}_{i},{\tilde{x}}_{k})=1-d({\tilde{x}}_{i},{\tilde{x}}_{k})=1-\sqrt{\frac{{\left({\tilde{x}}_{i}{}^{L}-{\tilde{x}}_{k}{}^{L}\right)}^{2}+{\left({\tilde{x}}_{i}{}^{M}-{\tilde{x}}_{k}{}^{M}\right)}^{2}+{\left({\tilde{x}}_{i}{}^{T}-{\tilde{x}}_{k}{}^{T}\right)}^{2}}{3}}$$

**Theorem** **2.** *The degree of triangular fuzzy similarity satisfies the following properties:*

*(1)*  ${\tilde{S}}_{TF}({\tilde{x}}_{i},{\tilde{x}}_{k})={\tilde{S}}_{TF}({\tilde{x}}_{k},{\tilde{x}}_{i})$.
*(2)*  ${\tilde{S}}_{TF}({\tilde{x}}_{i},{\tilde{x}}_{k})=1$*if*${\tilde{x}}_{i}={\tilde{x}}_{k}$*, and*${\tilde{S}}_{TF}({\tilde{x}}_{i},{\tilde{x}}_{k})=0$*if*${\tilde{x}}_{i}=[0,0,0]$*and*${\tilde{x}}_{k}=[1,1,1]$*or*${\tilde{x}}_{i}=[1,1,1]$*and*${\tilde{x}}_{k}=[0,0,0]$.

**Proof.**  According to Definition 5, (1) obviously holds.

(2) Since $0\leq {\tilde{S}}_{TF}({\tilde{x}}_{i},{\tilde{x}}_{k})\leq 1$, when ${\tilde{x}}_{i}={\tilde{x}}_{k}$, i.e., ${\tilde{x}}_{i}{}^{L}={\tilde{x}}_{k}{}^{L}$, ${\tilde{x}}_{i}{}^{M}={\tilde{x}}_{k}{}^{M}$,${\tilde{x}}_{i}{}^{T}={\tilde{x}}_{k}{}^{T}$, we have $d({\tilde{x}}_{i},{\tilde{x}}_{k})=0$, so ${\tilde{S}}_{TF}({\tilde{x}}_{i},{\tilde{x}}_{k})=1$. Similarly, since ${\tilde{x}}_{i}{}^{L},{\tilde{x}}_{i}{}^{M},{\tilde{x}}_{i}{}^{T}\in [0,1]$ and ${\tilde{x}}_{k}{}^{L},{\tilde{x}}_{k}{}^{M},{\tilde{x}}_{k}{}^{T}\in [0,1]$, $d({\tilde{x}}_{i},{\tilde{x}}_{k})\in [0,1]$. When ${\tilde{S}}_{TF}({\tilde{x}}_{i},{\tilde{x}}_{k})=0$, $d({\tilde{x}}_{i},{\tilde{x}}_{k})=1$. In this case, we can obtain ${\tilde{x}}_{i}=[0,0,0]$ and ${\tilde{x}}_{k}=[1,1,1]$ or ${\tilde{x}}_{i}=[1,1,1]$ and ${\tilde{x}}_{k}=[0,0,0]$. □

#### 4.2.2. Construction of the 3WD Model

**Definition** **6.** *Let U be the universe and A be the set of attributes. The triangular fuzzy similarity between any object*$x_{i}$*and*$x_{k}$*in U is*${\tilde{S}}_{SF}({\tilde{x}}_{i},{\tilde{x}}_{k})$*. If there is a threshold*δ*, then the*δ*-level classes of*x∈U*with respect to*${\tilde{S}}_{SF}({\tilde{x}}_{i},{\tilde{x}}_{k})$*are defined as follows (Equation (18)):*

(18) $$\begin{array}{l} {\left[{\tilde{S}}_{SF}^{\delta }\right]}_{s}=\left\{x\in U|{\tilde{S}}_{SF}({\tilde{x}}_{i},{\tilde{x}}_{k})>\delta \right\}\\ {\left[\;{\tilde{S}}_{SF}^{\delta }\right]}_{g}=\left\{x\in U|{\tilde{S}}_{SF}({\tilde{x}}_{i},{\tilde{x}}_{k})\leq \delta \right\} \end{array}$$

*where*${\left[\;{\tilde{S}}_{SF}^{\delta }\right]}_{s}$*and*${\left[\;{\tilde{S}}_{SF}^{\delta }\right]}_{g}$*are triangular fuzzy similarity classes of positive and negative fields, respectively. Specifically, the objects in*${\left[\;{\tilde{S}}_{SF}^{\delta }\right]}_{s}$*have the smallest degree of information difference and the largest triangular fuzzy similarity on the*δ*-level while the objects in*${\left[\;{\tilde{S}}_{SF}^{\delta }\right]}_{g}$*are the opposite to*${\left[\;{\tilde{S}}_{SF}^{\delta }\right]}_{s}$.

Suppose X⊆U is a given goal concept and $\Omega =\{{\left[\;{\tilde{S}}_{SF}^{\delta }\right]}_{s},{\left[\;{\tilde{S}}_{SF}^{\delta }\right]}_{g}\}$ is the set of states, which represents object *x* in the δ-level similarity domain ${\left[\;{\tilde{S}}_{SF}^{\delta }\right]}_{s}$ or *x* in the δ-level negative similarity domain ${\left[\;{\tilde{S}}_{SF}^{\delta }\right]}_{g}$. $\Gamma =\{a_{P},a_{B},a_{N}\}$ is the set of actions, where $a_{P}$ means acceptance, $a_{B}$ means delay, and $a_{N}$ means rejection. According to reference [75], the losses caused by actions taken in different states are shown in Table 1.

When the object $x\in {\left[\;{\tilde{S}}_{SF}^{\delta }\right]}_{s}$, $\lambda_{PP}$, $\lambda_{BP}$ and $\lambda_{NP}$ represent the loss of acceptance, delay and rejection decisions, respectively. Analogously, $\lambda_{PN}$, $\lambda_{BN}$ and $\lambda_{NN}$ represent the corresponding decision loss cost when $x\in {\left[\;{\tilde{S}}_{SF}^{\delta }\right]}_{g}$. Without any loss of generality, when $x\in {\left[\;{\tilde{S}}_{SF}^{\delta }\right]}_{s}$, we assume that the correct acceptance cost is less than the delay decision cost and less than the corresponding wrong acceptance cost, namely $\lambda_{PP}<\lambda_{BP}<\lambda_{NP}$. Similarly, when misclassified, we have $\lambda_{NN}<\lambda_{BN}<\lambda_{PN}$. Therefore, the expected losses $R(a_{•}|x)(\cdot \in \{P,B,N\})$ of object *x* under the above three decision actions are as follows (Equation (19)):

(19) $$\begin{array}{l} R(a_{P}|x)=\lambda_{PP}\mathrm{Pr}({\left[\;{\tilde{S}}_{SF}^{\delta }\right]}_{s}|x)+\lambda_{PN}\mathrm{Pr}({\left[\;{\tilde{S}}_{SF}^{\delta }\right]}_{g}|x),\\ R(a_{B}|x)=\lambda_{BP}\mathrm{Pr}({\left[\;{\tilde{S}}_{SF}^{\delta }\right]}_{s}|x)+\lambda_{BN}\mathrm{Pr}({\left[\;{\tilde{S}}_{SF}^{\delta }\right]}_{g}|x),\\ R(a_{N}|x)=\lambda_{NP}\mathrm{Pr}({\left[\;{\tilde{S}}_{SF}^{\delta }\right]}_{s}|x)+\lambda_{NN}\mathrm{Pr}({\left[\;{\tilde{S}}_{SF}^{\delta }\right]}_{g}|x)\ldots \end{array}$$

where $\;\mathrm{Pr}({\left[\;{\tilde{S}}_{SF}^{\delta }\right]}_{s}|x)=\;P(\;{\tilde{S}}_{SF}^{\delta }|x)$ and $\mathrm{Pr}({\left[\;{\tilde{S}}_{SF}^{\delta }\right]}_{g}|x)=1-\;P(\;{\tilde{S}}_{SF}^{\delta }|x)$ are the probabilities that object *x* belongs to a similar state of the δ-level positive or negative domain. By introducing Bayesian minimum risk decision theory, we have (Equation (20)):

(20) $$\begin{array}{l} (P)\;\lambda_{PP}P({\tilde{S}}_{SF}^{\delta }|x)+\lambda_{PN}(1-P({\tilde{S}}_{SF}^{\delta }|x))\leq \lambda_{BP}P({\tilde{S}}_{SF}^{\delta }|x)+\lambda_{BN}(1-P({\tilde{S}}_{SF}^{\delta }|x))\;and\;\\ \;\;\;\;\;\;\;\lambda_{PP}P({\tilde{S}}_{SF}^{\delta }|x)+\lambda_{PN}(1-P({\tilde{S}}_{SF}^{\delta }|x))\leq \lambda_{NP}P({\tilde{S}}_{SF}^{\delta }|x)+\lambda_{NN}(1-P({\tilde{S}}_{SF}^{\delta }|x))\\ (B)\;\;\lambda_{BP}P({\tilde{S}}_{SF}^{\delta }|x)+\lambda_{BN}(1-P({\tilde{S}}_{SF}^{\delta }|x))\leq \lambda_{PP}P({\tilde{S}}_{SF}^{\delta }|x)+\lambda_{PN}(1-P({\tilde{S}}_{SF}^{\delta }|x))\;and\;\\ \;\;\;\;\;\;\;\lambda_{BP}P({\tilde{S}}_{SF}^{\delta }|x)+\lambda_{BN}(1-P({\tilde{S}}_{SF}^{\delta }|x))\leq \lambda_{NP}P({\tilde{S}}_{SF}^{\delta }|x)+\lambda_{NN}(1-P({\tilde{S}}_{SF}^{\delta }|x))\\ (N)\;\;\lambda_{NP}P({\tilde{S}}_{SF}^{\delta }|x)+\lambda_{NN}(1-P({\tilde{S}}_{SF}^{\delta }|x))\leq \lambda_{PP}P({\tilde{S}}_{SF}^{\delta }|x)+\lambda_{PN}(1-P({\tilde{S}}_{SF}^{\delta }|x))\;and\;\\ \;\;\;\;\;\;\;\lambda_{NP}P({\tilde{S}}_{SF}^{\delta }|x)+\lambda_{NN}(1-P({\tilde{S}}_{SF}^{\delta }|x))\leq \lambda_{BP}P({\tilde{S}}_{SF}^{\delta }|x)+\lambda_{BN}(1-P({\tilde{S}}_{SF}^{\delta }|x)) \end{array}$$

Furthermore, form Equations (19) and (20), we can obtain (Equation (21)):

(21) $$\begin{array}{l} (P^{\prime })\;If\;P({\tilde{S}}_{SF}^{\delta }|x)\geq \alpha \;,\;then\;x\in Pos(X),\\ (B^{\prime })\;If\;\beta <P({\tilde{S}}_{SF}^{\delta }|x)<\alpha ,\;then\;x\in Bnd(X),\\ (N^{\prime })\;If\;P({\tilde{S}}_{SF}^{\delta }|x)\leq \beta \;,\;then\;x\in Neg(X)\ldots \end{array}$$

where (Equation (22))

(22) $$\alpha =\frac{\lambda_{PN}-\lambda_{BN}}{\left(\lambda_{PN}-\lambda_{BN})+(\lambda_{BP}-\lambda_{PP}\right)},\beta =\frac{\lambda_{BN}-\lambda_{NN}}{\left(\lambda_{BN}-\lambda_{NN})+(\lambda_{NP}-\lambda_{BP}\right)}.$$

In the Algorithm 1, we first measure the degree of information difference for each object according to any two attributes in the target domain (line 1 and line 2). On this basis, the triangular fuzzy similarity of each object can be calculated (line 3). It is worth noting that we can obtain triangular fuzzy similarity at different levels by adjusting the threshold parameter δ. Furthermore, the triangular fuzzy similarity is regarded as the cost loss of different classification decisions, and the final decision is implemented by comparing with the decision thresholds α and β (line 4). 

In addition, the higher the value of δ is, the greater the triangular fuzzy similarity between objects is. On the one hand, since ${\left[\;{\tilde{S}}_{SF}^{\delta }\right]}_{s}=\left\{x\in U|{\tilde{S}}_{SF}({\tilde{x}}_{i},{\tilde{x}}_{k})>\delta \right\},$${\left[\;{\tilde{S}}_{SF}^{\delta }\right]}_{g}=\left\{x\in U|{\tilde{S}}_{SF}({\tilde{x}}_{i},{\tilde{x}}_{k})\leq \delta \right\}$, by changing the parameter δ, we can obtain the triangular fuzzy similarity of objects in the target domain at different levels. One the other hand, the values of ${\left[\;{\tilde{S}}_{SF}^{\delta }\right]}_{s}$ and ${\left[\;{\tilde{S}}_{SF}^{\delta }\right]}_{g}$ will directly affect the values of threshold α and β. In order to visualize the impact of the final result and the threshold, we have shown it in detail in Section 6.1.
**Algorithm 1** Three-way decision model based on the triangular fuzzy similarity**Input:** target domain data $X_{t}$, threshold δ, α and β.**Output:** positive region object set Pos(X), negative region object set Neg(X), boundary region object set Bnd(X).**1: BEGIN****2:** Calculate the degree of information difference $ID_{i}(a_{j},a_{k})$ of each object in the target domain under any two attributes according to Equation (15).**3:** Calculate the triangular fuzzy similarity ${\tilde{S}}_{TF}({\tilde{x}}_{i},{\tilde{x}}_{k})$ between any two objects in the target domain using Equation (17).**4:** According to Equation (21), divide the target domain $X_{t}$ into three domains.**5: END BEGIN**

### 4.3. Adaptation Via Iterative Refinement

In this section, we integrate the methods presented in Section 4.1 and Section 4.2 and finally realize unsupervised domain adaptation to the conditional distribution of cross-domain data by introducing the label propagation algorithm. Specifically, we first obtain the initial pseudolabel ${\hat{y}}_{T}$ of the target domain according to joint distribution adaptation, then obtain the set of boundary objects of the target domain according to the three-way decision model proposed in Section 4.2 and place these objects into objects to be classified. Once the above ${\hat{y}}_{T}$ and Bnd(X) are obtained, we effectively set a semisupervised setting for the target domain data. Following [29], we use the label propagation algorithm to discriminate the boundary objects in the target domain and update ${\hat{y}}_{T}$. Algorithm 2 summarizes our proposed method. Algorithm 2—which in addition to the initial stage, we only adapt to the marginal distribution—and the subsequent steps consider both the marginal distribution and the conditional distribution. In addition, the accuracy of the labels in the target domain is gradually improved as the cross domain distribution differences decrease. In the following experiments, we will show that the proposed method converges to the optimal solution in a finite number of iterations and further prove the effectiveness of the proposed method.
**Algorithm 2** Our Proposed Model**Input:** source domain data $X_{s}$, target domain data $X_{t}$, labels $y_{S}$ of source domain data, threshold δ, α and β**Output:**$y_{T}$ as labels of target domain data**1: BEGIN****2:** Initialize $D_{t}^{\left(c\right)}$ as Null**3: while** not converged **do****4:**   (1) W ← Distribution adaptation $\left(D_{t}^{\left(c\right)}\ldots {\hat{y}}_{T}\right)$ in Equation (14) and let $Z_{s}=W^{T}X_{s}$ and $Z_{t}=W^{T}X_{t}$**5:**    (2) Assign ${\hat{y}}_{T}$ using classifiers trained by $Z_{s}$**6:**    (3) Obtain Bnd(X) in Algorithm 1**7:**     (4) $\left(D_{t}^{\left(c\right)}\ldots {\hat{y}}_{T}\right)$ ← execute label propagation algorithm**8: End while****9:**$y_{T}$←${\hat{y}}_{T}$**10: END BEGIN**

## 5. Experiments

### 5.1. Materials

#### 5.1.1. Data Acquisition

In order to verify the effectiveness of our proposed method and compare this method with the existing research, our experimental data are obtained from the publicly accessible ABIDE dataset. ABIDE is a multisite platform that has aggregated functional and structural brain imaging data collected from 17 different laboratories around the world, which including 539 ASD patients and 573 neurotypical controls. All subjects had corresponding resting-state fMRI images and phenotypic information such as age and gender. More details on the data collection, exclusion criteria, and scan parameters are available on the ABIDE website, namely, http://fcon_1000.projects.nitrc.org/indi/abide/, (accessed on 8 October 2020). As different sites have different numbers of limited samples, we use the data from three different sites, including NYU, UM and USM, each with more than 50 subjects and using different fMRI protocols. Specifically, there were 343 subjects, including 159 ASD patients and 184 neurotypical controls. Detailed demographic information of the subjects is listed in Table 2. In Table 2, m ± std and M/F are short for mean ± standard deviation and male/female, respectively. In each site, we used the two-sample t-test to evaluate the differences in age between the two groups and no significant differences was observed between the control group and the ASD group, i.e., *p* = 0.42 (NYU), *p* = 0.31 (USM), *p* = 0.34 (UM). Since the subjects across different sites follow different distributions, it is necessary to perform domain adaptation. In the experiments, we use A→B to denote the knowledge transfer from source domain A to target domain B. We construct a total of six tasks: NYU→USM, NYU→UM, USM→NYU, USM→UM, UM→NYU, and UM→USM.

#### 5.1.2. Data Pre-Processing

To ensure replicability, each rs-fMRI datapoint used in this research was provided by the Preprocessed Connectome Project initiative and preprocessed by using the Data Processing Assistant for Resting-State fMRI (DPARSF) software [76]. The image preprocessing steps are listed as follows. (1) Remove the first 10 time points, (2) conduct slice timing correction, and (3) conduct head motion realignment. (4) Next, image standardization was performed by normalizing the functional images into the echo planar imaging (EPI) template, followed by (5) spatial smoothing, (6) removing the linear trend, (7) temporal filtering, and (8) removing covariates. Subsequently, the brain was divided into 90 regions of interest (ROIs) based on the Automatic Anatomical Labelling (AAL) [77] atlas, and the average time series of each ROI was extracted. Then, for each subject, we obtained a 90 × 90 functional connectivity symmetric matrix, where each element represents the Pearson correlation coefficient between a pair of ROIs. Finally, we convert the upper triangle into a 4005 (90 × 89/2)-dimensional feature vector to represent each subject.

### 5.2. Competing Methods

We compared the performance of our method with the following state-of-the-art machine learning models, including one baseline method and three representation-based methods.

Baseline: In this study, we use a support vector machine (SVM) as the base classifier, which is widely used in the field of neuroimaging [11]. Specifically, we specify site data as the source domain, directly train an SVM model using the original features on it, and then use the rest of the site data as the target domain to test the classifier we have trained. In the SVM classifier, we applied a linear kernel and searched the margin penalty using the grid-search strategy from the range of [2^−5^, 2^−4^…, 2^4^, 2^5^] via cross-validation.

Transfer component analysis (TCA) **[36]**: This is a general feature transformation method that reduces the difference in the marginal distribution between different domains by learning the transfer components between domains in RKHS.

Joint distribution adaptation (JDA) **[26]**: The JDA approach reduces both the marginal distribution and conditional distribution between different domains.

Domain adaptation with label and structural consistency (DALSC) [29]: DALSC is an unsupervised domain adaptation method that uses the structural information of the target domain to improve the performance of the model while adjusting the marginal distribution and conditional distribution between domains.

### 5.3. Experimental Setup

In this work, we use 5-fold cross-validation to evaluate the performance of each method. For our method, we set δ=0.3, β is searched in {0.5,0.55,⋯,0.85,0.9}, α is searched in {0.55,0.6,⋯,0.9,0.95}, and α>β. In addition, to evaluate the classification performance, we calculated the true positives (TPs), false positives (FPs), true negatives (TNs), and false negatives (FNs) for the classification by comparing the classified labels and gold-standard labels. Then, six evaluation metrics on test data, including the classification accuracy (*ACC*), sensitivity (*SEN*), specificity (*SPE*), balanced accuracy (*BAC*), positive predictive value (*PPV*) and negative predictive value (*NPV*), are utilized. These metrics can be computed as follows (Equation (23)):

(23) $$\begin{array}{l} ACC=\left(TP+TN\right)/\left(TP+FN+TN+FP\right),SEN=TP/\left(TP+FN\right)\\ SPE=TN/\left(TN+FP\right),BAC=\left(SEN+SPE\right)/2\\ PPV=TP/\left(TP+FP\right),NPV=TN/\left(TN+FN\right) \end{array}$$

For these metrics, higher values indicate better classification performance.

### 5.4. Results on ABIDE with Multisite fMRI Data

In this section, we present the experimental results of the proposed method and several other comparative methods on six tasks. Note that data from each site can be used as the source domain while the data from other sites can be used as the target domain. For the three domain adaptation methods (i.e., TCA, JDA, and DALSC) and our proposed method, an unsupervised adaptive experimental setup is adopted, which has no label information of the target domain to be utilized in the prediction process. The classification performance results of various methods are shown in Table 3. From Table 3, we can make the following three observations.

First, in terms of accuracy, the domain adaptive method based on feature representation is better than the direct use of the SVM classifier to predict the target domain.

Second, the TCA method in the domain adaptation method has the worst classification result because it only considers the marginal distribution.

Finally, the experimental results show that the classification accuracy of the proposed method is better than the existing domain adaptive methods (such as TCA, JDA and DALSC) in six tasks, and it also has good performance in SEN, SPE, BAC and other indicators.

## 6. Discussion

In this section, we first analyze the influence of the parameters in the proposed method on the algorithm performance and then compare the proposed method with other state-of-the-art methods.

### 6.1. Parameter Analysis

**Figure 1:**
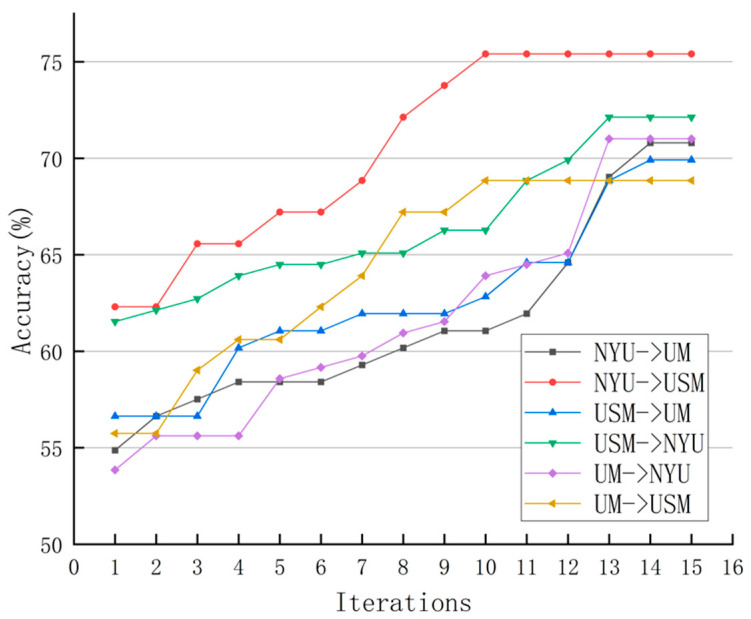
Classification accuracy versus the number of iterations on six domain pairs.

We first analyze the impact of the number of iterations on the performance of the proposed method. As mentioned in Section 4.3, for domain adaptation, we solve the proposed model iteratively. In order to evaluate its convergence, Figure 1 shows the change in algorithm accuracy as the number of iterations increases on the six tasks. It can be seen from Figure 1 that the classification accuracy of each task is gradually improved with the increase in the number of iterations. This indicates that our model learned an invariant data distribution among domains/sites after multiple iterations. The figure shows that the accuracy rate converges in 10–15 iterations.

**Figure 2:**
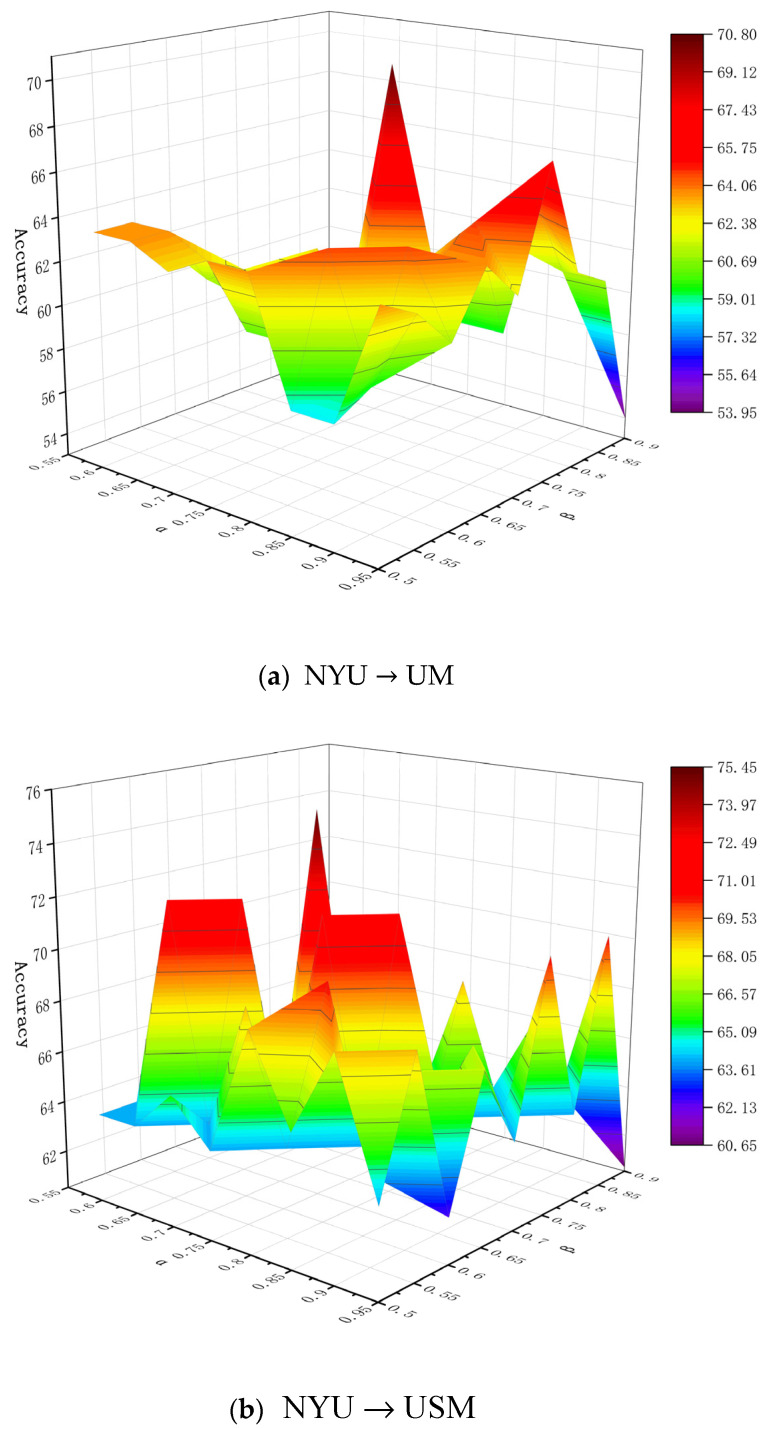

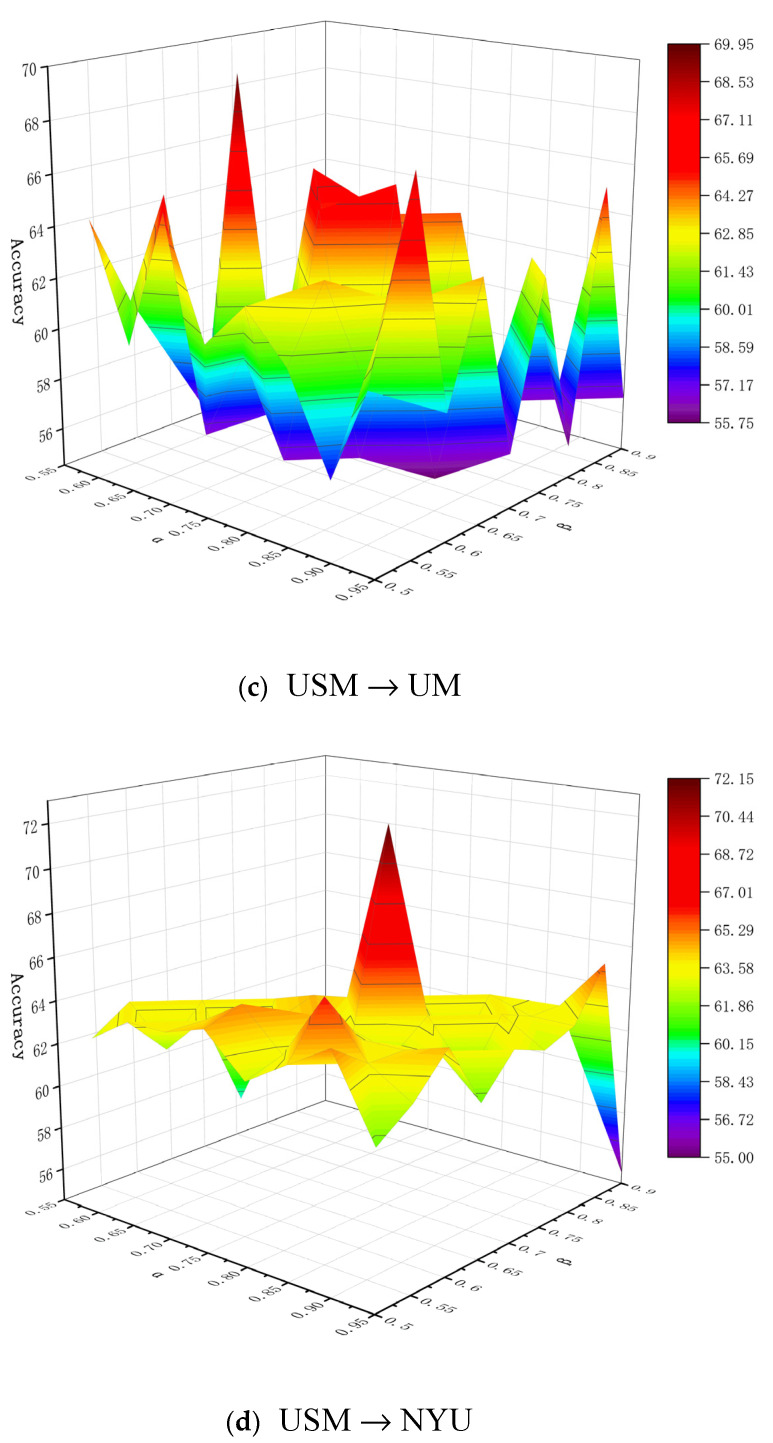

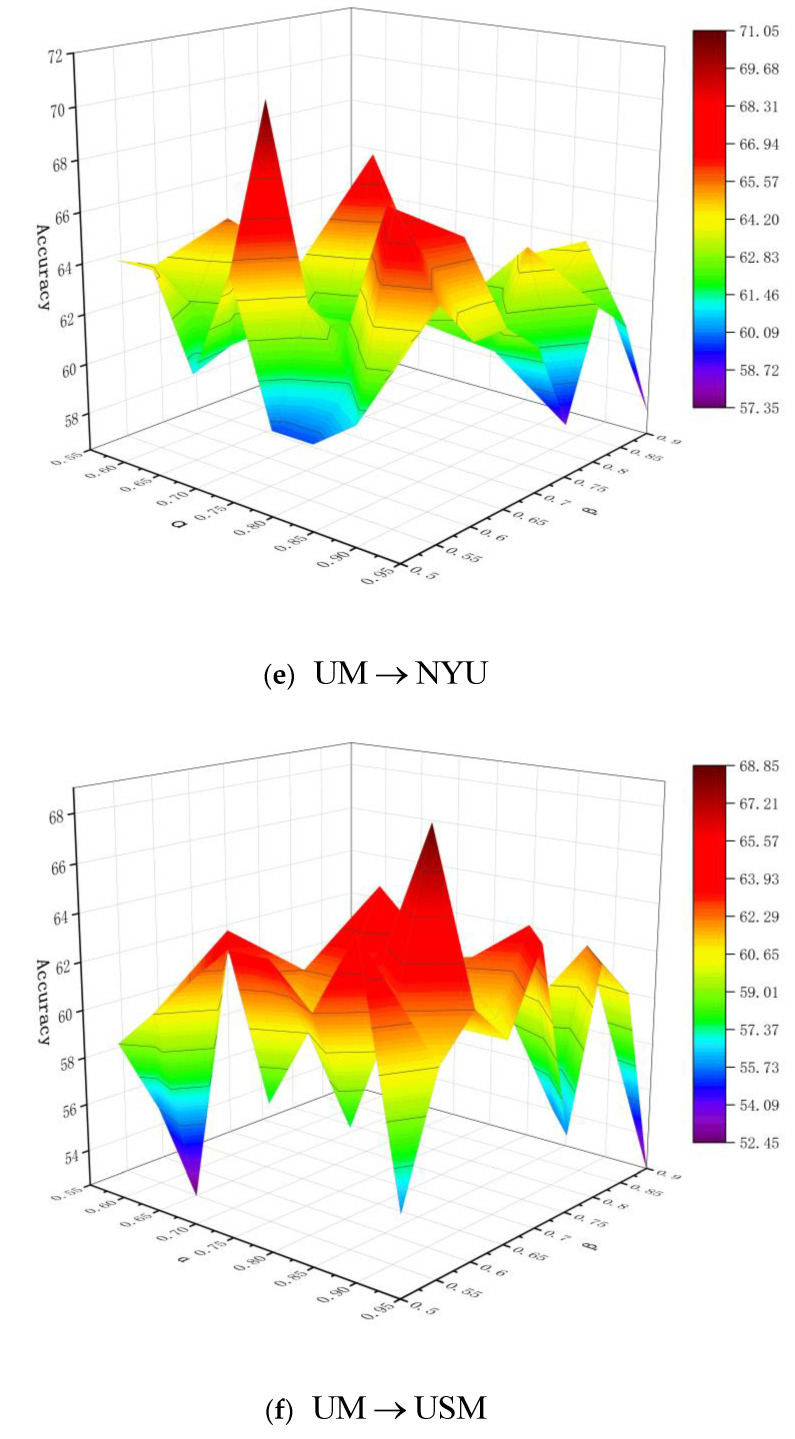
Classification accuracies with respect to different parameter values of α and β on six domain pairs (**a**) NYU→UM; (**b**) NYU→USM; (**c**) USM→UM; (**d**) USM→NYU; (**e**) UM→NYU; (**f**) UM→USM.

In addition, the values of α and β involved in the experiment represent different decision risk cost levels, and their slight differences may induce different decision results. Without any loss of generality, in order to obtain more suitable parameters, we analyze the influence of different threshold parameters on the performance of the proposed method. Specifically, in order to evaluate the method’s convergence, we conducted comparison experiments at different levels on the six tasks, and the final results are shown in Figure 2. The figures show that the accuracy of the algorithm changes as the threshold changes; and although the degree of fluctuation of the accuracy is different under different (α,β), it will eventually converge. It can be seen from Figure 2 that the optimal values of (α,β) under six tasks NYU→UM, NYU→USM, USM→UM, USM→NYU, UM→NYU and UM→USM are (0.8, 0.7), (0.75, 0.65), (0.7, 0.6), (0.8, 0.7), (0.75, 0.55), (0.9, 0.6), respectively. Furthermore, it can be observed from Figure 2 that when given smaller β and larger α, the classification accuracy of the six tasks is relatively low. This shows that smaller β and larger α result in more samples from the target domain being divided into the boundary region. More boundary objects increase the uncertainty information when implementing the label propagation algorithm, which leads to the decline of classification performance.

### 6.2. Comparison with State-of-the-Art Methods

To further verify the effectiveness of our proposed method, we also compare it with six other advanced methods (including the deep learning method) using the rs-fMRI data in the ABIDE database. Since only a few research papers have reported their average classification results among different sites, we only list the classification results on the NYU site in Table 4. In addition, we list the details of each method in Table 4, including the classifier and the type of feature. It is worth noting that in the research of [14,17], they selected a part of the samples from each site in proportion as the training set and then used the trained deep learning model to predict the NYU site directly.

As Table 4 shows, the proposed method achieves 72.13% and 71.01% classification accuracy, respectively in the two tasks with NYU as the target domain, which is better than the models proposed in other research papers. In terms of feature type and feature dimension, this paper uses AAL atlas to divide brain regions, and obtains the original feature vector with the smallest dimension. In addition, although the sGCN, DAE and DANN are three deep learning methods, our proposed method still has a better classification effect. There may be two reasons for this. (1) Training a robust deep learning model usually requires a large number of samples. However, for multisite ASD recognition, although the data from each site can be fused together to generate a larger data set, these samples are still insufficient to train a reliable deep neural network. (2) The overfitting problem usually occurs when a deep neural network processes data with noise. In fact, fMRI data usually contain a large amount of noise information, which limits the generalization ability of the trained neural network.

## 7. Conclusions

In this paper, we propose a novel domain adaptation method for ASD identification with rs-fMRI data. Specifically, we introduce a three-way decision model based on triangular fuzzy similarity and divide the objects in the target domain with coarse granularity. Then, a label propagation algorithm is used to make secondary decisions on boundary region objects so as to improve the performance of ASD diagnosis based on cross-site rs-fMRI data. We conduct extensive experiments on the ABIDE dataset based on multisite data to verify the convergence and robustness of the proposed algorithm. Compared with several state-of-the-art methods, the experimental results show that the proposed method has better classification performance.

Although the classification results of our proposed method based on cross-site ASD diagnosis are significantly improved compared with the existing domain adaptation methods based on feature distribution, the following technical problems need to be considered in the future. First, although the proposed method can alleviate data heterogeneity between source and target domains, the input fMRI features are still unfiltered original high-dimensional features. However, the original high-dimensional features may have redundant features, which will reduce the performance of the model. Therefore, in the future, we will study how to combine feature selection with our methods for ASD diagnosis. Second, in this paper, we only take the functional connectivity matrix of rs-fMRI data as the feature representation of each subject without considering the network topology information. In future research, we will consider the fusion of functional brain network topology data to provide more valuable discriminant information for ASD diagnosis. Finally, in order to obtain more valuable structured information of the target domain, we will consider combining multigranularity rough sets to further improve the model performance in the future.

## Figures and Tables

**Table 1:** Cost function matrix.

Action	Cost Function	
	${\left[{\tilde{S}}_{SF}^{\delta }\right]}_{s}$	${\left[{\tilde{S}}_{SF}^{\delta }\right]}_{g}$
$a_{P}$	$\lambda_{PP}$	$\lambda_{PN}$
$a_{B}$	$\lambda_{BP}$	$\lambda_{BN}$
$a_{N}$	$\lambda_{NP}$	$\lambda_{NN}$

**Table 2:** Demographic information of the studied subjects from three imaging sites in the ABIDE database. The age values are denoted as the mean ± standard deviation. M/F: male/female.

Site	ASD		Normal Control	
	Age (m ± std)	Gender (M/F)	Age (m ± std)	Gender (M/F)
NYU	14.92 ± 7.04	64/9	15.75 ± 6.23	70/36
USM	24.59 ± 8.46	38/0	22.33 ± 7.69	23/0
UM	13.85 ± 2.29	39/9	15.03 ± 3.64	49/16

**Table 3:** Performance of five different methods in ASD classification on the multisite ABIDE database. The number in bold indicates the best result achieved under a certain metric.

Task	Method	ACC (%)	SEN (%)	SPE (%)	BAC (%)	PPV (%)	NPV (%)
**NYU** **→UM**	Baseline	54.87	49.23	62.5	55.87	64	47.62
	TCA	62.83	58.46	68.75	63.61	71.69	55.00
	JDA	64.50	66.67	61.64	64.16	69.57	58.44
	DALSC	64.60	56.92	**75.00**	65.96	75.51	56.25
	Ours	**70.80**	**72.31**	68.75	**70.53**	**75.81**	**64.71**
**NYU** **→USM**	Baseline	67.21	78.26	60.53	69.39	54.55	82.14
	TCA	68.85	82.61	60.53	71.57	55.88	85.19
	JDA	70.49	86.96	60.53	73.74	57.14	88.46
	DALSC	72.13	73.91	**71.05**	72.48	60.71	81.81
	Ours	**75.41**	**91.30**	65.79	**78.55**	**61.76**	**92.59**
**USM** **→UM**	Baseline	57.52	35.38	**87.50**	61.44	**79.31**	50.00
	TCA	58.41	38.46	85.42	61.94	78.13	50.62
	JDA	61.06	61.54	60.42	60.98	67.80	53.70
	DALSC	64.60	73.85	52.08	62.96	67.61	59.52
	Ours	**69.91**	**76.92**	60.42	**68.67**	72.46	**65.91**
**USM** **→NYU**	Baseline	53.25	35.42	76.71	56.06	66.67	47.46
	TCA	57.39	40.63	**79.45**	60.04	**72.22**	50.43
	JDA	60.36	64.58	54.79	59.69	65.26	54.05
	DALSC	63.91	65.63	61.64	63.63	69.23	57.69
	Ours	**72.13**	**78.26**	68.42	**73.34**	60.00	**83.87**
**UM** **→NYU**	Baseline	58.58	83.33	26.03	54.68	59.70	54.29
	TCA	61.54	82.29	34.25	58.27	62.20	59.50
	JDA	63.31	82.29	38.35	60.32	63.71	62.22
	DALSC	64.49	**92.70**	27.39	60.05	62.68	74.07
	Ours	**71.01**	90.63	**45.21**	**67.92**	**68.50**	**78.57**
**UM** **→USM**	Baseline	54.09	78.26	39.47	58.87	43.90	75.00
	TCA	60.66	73.91	52.63	63.27	48.57	76.92
	JDA	60.66	78.26	50.00	64.13	48.65	79.17
	DALSC	57.38	73.91	47.37	60.64	45.95	75.00
	Ours	**68.85**	**82.61**	**60.53**	**71.57**	**55.88**	**85.19**

**Table 4:** Comparison with state-of-the-art methods for ASD identification using rs-fMRI ABIDE data on the NYU site. HOA: Harvard Oxford Atlas. GMR: grey matter ROIs, and AAL: anatomical automatic labelling. CC200: Craddock 200. sGCN: siamese graph convolutional neural network. FCA: functional connectivity analysis. DAE: denoising autoencoder. DANN: deep attention neural networks.

Method	Feature Type	Feature Dimension	Classifier	ACC (%)
sGCN + Hing Loss [14]	HOA	111 × 111	K-Nearest Neighbor (KNN)	60.50
sGCN + Global Loss [14]	HOA	111 × 111	KNN	63.50
sGCN + Constrained Variance Loss [14]	HOA	111 × 111	KNN	68.00
FCA [17]	GMR	7266 × 7266	*t*-test	63.00
DAE [16]	CC200 Atlas	19,900	Softmax Regression	66.00
DANN [78]	AAL	6670	Deep neural network	70.90
Ours	AAL	4005	SVM	72.13/71.01

## Data Availability

The data presented in this study are available on request from the corresponding author.
